# ASO Author Reflections: What is the Current Role for Palliative Care Within Surgical Oncology?

**DOI:** 10.1245/s10434-025-17191-2

**Published:** 2025-03-20

**Authors:** Amanda K. Walsh, Jordan M. Cloyd

**Affiliations:** Division of Surgical Oncology, Department of Surgery, The Ohio State University Wexner Medical Center, Columbus, OH USA

## Past

Palliative care (PC) is an interdisciplinary practice aimed at improving quality of life (QoL) for patients with serious illnesses and their families by prioritizing symptom control, psychosocial support, and treatment approaches consistent with patients’ values. Historically, PC has been reserved for end-of-life care. More recent evidence suggests that it serves an important role throughout treatment, particularly for patients with complex needs, such as those with a diagnosis of cancer.^1^ In fact, early PC leads to improved outcomes for patients with advanced cancer, which has led to the recommendation for early integration of PC in existing guidelines for patients with newly diagnosed advanced cancer.^1,2^ However, the role of PC for patients with early-stage cancer undergoing curative-intent cancer surgery is less clear and the evidence more sparse. As such, this study sought to systematically assess existing literature on the role of PC in surgical oncology.

## Present

This systematic review identified 14 studies including 2 cross-sectional studies of physician perspectives, 4 cohort studies, 5 qualitative studies, 1 non-randomized trial, and 2 randomized controlled trials (RCTs).^3^ Both RCTs randomized patients undergoing elective major abdominal cancer surgery to either perioperative PC consultation or usual care. Although neither RCT found significant differences in their primary endpoint, health-related QoL, the secondary analyses did highlight some ways that this patient population could benefit from PC interventions as well as future opportunities to improve PC integration. Several cohort studies identified low rates of PC utilization overall as well as disparities in PC referral based on clinical and sociodemographic factors. One cohort study found an increase in advance directive designation before cancer surgery when PC is integrated into a surgical oncology clinic. Although the overall quality of the included studies was rated highly, several gaps in the available evidence for PC within surgical oncology were identified.

## Future

This systematic review summarized the existing literature on the role of specialty PC within surgical oncology, noting that based on the results of two large RCTs, the routine preoperative use of PC referral is not justified. Still, the supportive care needs of patients undergoing planned curative-intent surgery should not be ignored. Indeed, in addition to the psychosocial impact of a new cancer diagnosis and the physical side effects of the cancer in situ, significant challenges and stressors specific to the surgical experience, including postoperative pain, advance directive discussions, and the physical demands of surgical recovery, could benefit from PC involvement. Future studies therefore should focus on identifying subpopulations within surgical oncology for whom PC referral would be the most useful, for example, patients with a high preoperative symptom burden, those with challenging postoperative recovery, or patients whose planned operations are aborted unexpectedly.^4,5^ Further research also should explore the role of primary PC as an effective method of delivering patient-centered supportive care within surgical oncology. Because most surgeons receive little training in PC competencies, additional research will be needed to assess this alternative model of PC delivery. Taken together, despite the recent negative RCTs, there is ample opportunity to expand the role of PC within surgical oncology with the overall aim of supporting patient well-being and QoL while pursuing curative-intent oncologic goals.

## References

[1] Temel JS, Greer JA, Muzikansky A, Gallagher ER, Admane S, Jackson VA, et al. Early palliative care for patients with metastatic non-small cell lung cancer. *N Engl J Med.*. 2010;363:733–42. 20818875 10.1056/NEJMoa1000678

[2] Sanders JJ, Temin S, Ghoshal A, Alesi ER, Ali ZV, Chauhan C, et al. Palliative care for patients with cancer: ASCO guideline update. *J Clin Oncol.*. 2024;42:2336–57. 38748941 10.1200/JCO.24.00542

[3] Walsh AK, Guo MZ, Leuschner T, et al. The role of specialty palliative care in elective surgical oncology: a systematic review. *Ann Surg Oncol.*. 2025. 10.1245/s10434-025-17076-4. 40000563 10.1245/s10434-025-17076-4 PMC12049279

[4] Cloyd JM, Khatri R, Sarna A, Stevens L, Heh V, Dillhoff M, et al. Early palliative care following aborted cancer surgery: results of a prospective feasibility trial. *Ann Surg Open.*. 2024;5:e520. 39711683 10.1097/AS9.0000000000000520 PMC11661761

[5] Lilley EJ, Baig MP, Huma S, Cooper Z. Perioperative symptoms: a new frontier for surgical palliative care. *Ann Surg.*. 2021;274:e80–1. 10.1097/SLA.0000000000004698. 33351470 10.1097/SLA.0000000000004698

